# Assessment of quality of life (QOL) in cancer patients attending oncology unit of a Teaching Hospital in Bangladesh

**DOI:** 10.1002/cnr2.1829

**Published:** 2023-05-19

**Authors:** Nazmul Islam, Alok Atreya, Samata Nepal, Kazi Jashim Uddin, Md. Rashed Kaiser, Ritesh G. Menezes, Savita Lasrado, Muhammad Abdullah‐Al‐Noman

**Affiliations:** ^1^ Department of Oncology Delta Medical College & Hospital Dhaka Bangladesh; ^2^ Department of Forensic Medicine Lumbini Medical College Palpa Nepal; ^3^ Department of Community Medicine Lumbini Medical College Palpa Nepal; ^4^ Department of Pathology, College of Medicine Imam Abdulrahman Bin Faisal University Dammam Saudi Arabia; ^5^ Department of Otorhinolaryngology and Head & Neck Surgery Father Muller Medical College Mangalore India; ^6^ Department of Radiation Oncology National institute of Cancer Research & Hospital Dhaka Bangladesh

**Keywords:** Bangladesh, cancer, EORTC QLQ‐C30, quality of life

## Abstract

**Background:**

The quality of life (QoL) of a cancer patient is their perception of their physical, functional, psychological, and social well‐being. QoL is one of the most important factors to consider when treating someone with cancer and during follow‐up. The aim of this study was to understand the state of QoL among cancer patients in Bangladesh and to determine the factors that affect it.

**Methods:**

This cross‐sectional study was conducted on 210 cancer patients who attended the oncology unit of Delta Medical College & Hospital, Dhaka during the period between 1 May 2022 and 31 August 2022. Data were collected using the Bengali version of the European Organization for Research and Treatment of Cancer (EORTC) questionnaire.

**Results:**

The study reported a high number of female cancer patients (67.6%), who were married, Muslims by religion, and non‐residents of Dhaka. Breast cancer was more common among women (31.43%), while lung and upper respiratory tract cancer was more prevalent among men (19.05%). The majority of the patients (86.19%) were diagnosed with cancer in the past year. The overall mean score for functional scales was higher for physical functioning (54.92) whereas it was lower for social functioning (38.89). The highest score on the symptom scale was for financial problems (63.02), while the lowest was for diarrhea (33.01). The overall QoL score of cancer patients in the study was 47.98 and it was lower for males (45.71) compared to females (49.10).

**Conclusions:**

The overall QoL was poor among Bangladeshi cancer patients compared to those in developed countries. A low QoL score was observed for social and emotional functions. Financial difficulty was the main reason behind the lower QoL score on the symptom scale.

## 1 INTRODUCTION

Bangladesh is a low‐income country in South Asia with a population of 165 158 616.^1^ Among the population, 20.5% live in poverty and 10.5% live in extreme poverty.^1^ In 2020, there were 156 775 new cases of cancer and 108 990 cancer‐related deaths reported in the country.^2^ Lung, prostate, and esophageal cancers were the eighth, tenth, and fourteenth leading causes of total mortality in men, while breast, cervical, leukemia/lymphoma, and other cancers were the eleventh, twelfth, thirteenth, and fifteenth leading causes of total mortality in women.^3^ Quality of life (QoL) is a critical parameter for follow‐up in cancer patients undergoing treatment and reflects their physical, functional, psychological, and social wellbeing.^4^ Considering QoL is essential when making a therapeutic decision as there are various treatment regimens for cancer.^4^

In 2022, the government planned to establish 18 000 community clinics and add 1400 additional beds to provide cancer care as there were fewer than 500 beds available in 2013.^2^, ^5^ This demonstrates the government's commitment to cancer care. However, providing cancer care is challenging and time‐consuming as it requires collaboration with various health care services, including pathology, radiology, surgical oncology, medical oncology, gynecology, and palliative care.

Cancer and its treatment result in excruciating misery for patients, both those who survive and those who succumb to the disease. Several studies have shown that cancer and its treatment can result in significant physical, emotional, and social distress in patients, negatively affecting their QoL. Moreover, the effectiveness of cancer treatment depends not only on increasing survival rates but also on how well it improves patients' overall well‐being.^4^ Therefore, assessing QoL is crucial in making treatment decisions, particularly in advanced stages of cancer, where the primary goal of therapy is to improve patients' feelings.^4^ However, the value of QoL measurement depends on their accuracy in capturing the key facets of patients' QoL, their reliability, responsiveness, and clinical relevance in terms of benefits and associated costs.^6^

Various QoL assessment tools are available, and the European Organization for Research and Treatment of Cancer (EORTC) QLQ‐C30 questionnaire is a reliable and valid method for assessing the QoL of cancer patients participating in trials and research where patient‐reported outcomes are collected.^7^ The EORTC QLQ‐C30 questionnaire has met all necessary criteria for validity, reliability, and sensitivity, making it a suitable method for assessing the QoL of cancer patients in various studies.^7^ This study aims to use this tool to assess the QoL of cancer patients in Bangladesh and to identify the factors affecting it. The information generated from this study will be essential for making informed therapeutic decisions, improving cancer patients' overall wellbeing, and strengthening cancer care delivery in Bangladesh and other low‐income countries facing similar challenges.

## 2 METHODS

The study proposal and the consent form were approved by the Ethics Committee of Delta Medical College Hospital, Dhaka (DLMCH/IEC/2022/1). The present study was conducted in the Oncology Unit of Delta Medical College & Hospital between 1 May 2022 and 31 August 2022. The expected number of new cancer patients visiting the department during the study period was 400. We calculate the sample size using the formula *n* = *Z* ^2^ (*p* × *q*)/*E* ^2^, where the population proportion (*p*) was 0.50, *p* − 1 (*q*) was 0.50, the 95% confidence level (*Z*) was 1.96, and the margin of error (*E*) was 0.05 for a population size (*N*) 400. The minimum sample size (*n*) calculated was 197.

### 2.1 Study tool

For the purpose of this study, we requested permission from the EORTC to use the Bengali version of their EORTC QLQ‐C30 questionnaire. EORTC provided the research tool and scoring manuals for the study. The 30‐item questionnaire covers 15 domains consisting of five functioning scales (physical functioning, social functioning, role functioning, emotional functioning, and cognitive functioning) and nine symptom scales (fatigue, pain, nausea/vomiting, dyspnea, sleep disturbances, appetite loss, diarrhea, constipation, and financial difficulties) and one global health status/quality of life scale.^7^ Scores closer to a maximum of 100 on the functioning and global health status/QoL scales indicate a higher level of QoL, while high scores on the symptom scales indicate a high burden of symptoms.^8^

### 2.2 Data collection

Data were collected 2 days a week, on Monday and Wednesday. This was due to the roster duty of the authors involved in data collection. The objective of the study was explained to the patients and informed consent was obtained from those who participated. All adult patients who visited to the outpatient clinic and all patients newly admitted to the inpatient clinic on those days were included in the study and were administered the questionnaire by the authors (NI, KJU and MRK) in person. Literate patients filled out the questionnaire by themselves. For illetrate patients, authors dictated the items in the questionnaire and recorded their responses. Patients who were interviewed for this study previously, those who could not provide consent (unconscious), patients with suspected cancer but without a confirmed report, and patients less than 18 years of age were excluded. Sociodemographic characteristics such as age at treatment, gender, marital status, religion, economic status, and education were obtained from the patients. Information on clinical status, such as the site of the primary tumor, the stage of the tumor, and the type of treatment, was recorded from the clinical documentation.

### 2.3 Data analysis

Data collected were entered into an Excel worksheet and exported to the Statistical Package for the Social Sciences (SPSS) v.21.0 (IBM Corp., Armonk, NY, USA) for analysis. Descriptive statistics (mean, standard deviation (SD), frequency and percentage) were used to describe the distribution of the type, stage, and treatment of the cancer. Categorical variables were expressed as percentage and compared using the Chi‐square test, with a *p*‐value of less than .05 considered statistically significant. All the underlying data for the present study is available without restriction.^9^

## 3 RESULTS

In the study 210 cancer patients were included, of whom 68 were men and 142 women with a male–female ratio of 1:2.09. The age of the patients ranged from 18 to 85 years with a mean age of 51.09 (13.22) years. Majority of the patients were Muslim by religion, unemployed and had an average family income (Table 1).

**TABLE 1:** Sociodemographic characteristics of the participants.

Characteristics	Frequency, *n* (%)
Gender	
Male	68 (32.4)
Female	142 (67.6)
Age range (in years)	
18–39	39 (18.6)
40–49	49 (23.3)
50–59	60 (28.6)
60–69	41 (19.5)
>70	21 (10.0)
Religion	
Muslim	192 (91.4)
Hindu	16 (7.6)
Buddhist	2 (1.0)
Address	
Dhaka residents	50 (23.81)
Non‐Dhaka residents	160 (76.19)
Level of education	
Illiterate	10 (4.8)
Primary	37 (17.6)
Secondary	49 (23.3)
Undergraduate	65 (31.0)
Postgraduate	49 (23.3)
Marital status	
Unmarried	15 (7.1)
Married	179 (85.2)
Divorced	4 (1.9)
Widowed	12 (5.7)
Employment	
Not working	76 (36.2)
Employed	68 (32.4)
Tradesman (self‐employed)	32 (15.2)
Retired	34 (16.2)
Economic status^a^	
Very good	15 (7.1)
Good	92 (43.8)
Average	103 (49.0)
Duration of hospitalization in last 30 days	
Not hospitalized	71 (33.8)
1–7 days	83 (39.5)
8–14 days	48 (22.9)
15–29 days	8 (3.8)
Health insurance status	
None	210 (100)

^a^ Participant's self‐reported status.

Almost 4/5th of the patients were diagnosed with cancer in the last year, and breast cancer was the most common type of cancer diagnosed in patients, followed by oropharyngeal cancer. Cancer‐related characteristics including the stage and treatment is depicted in Table 2.

**TABLE 2:** Cancer‐related characteristics of the patient.

Cancer related characteristics	Male	Female	*n* (%)
Primary diagnosis			
Breast cancer	0	66 (31.43)	66 (31.43)
Pharynx‐larynx‐oral cavity cancer	19 (9.05)	9 (4.29)	28 (13.3)
Lungs	21 (10.00)	3 (1.43)	24 (11.43)
Uterus‐cervix‐vagina cancer	0	19 (19.05)	19 (9.05)
Liver‐gallbladder‐pancreatic cancer	7 (3.33)	9 (4.29)	16 (7.62)
Stomach‐esophageal cancer	7 (3.33)	8 (3.81)	15 (7.14)
Colon‐rectal cancer	3 (1.43)	6 (2.86)	9 (4.29)
Ovarian cancer	0	9 (4.29)	9 (4.29)
Tongue	4 (1.90)	2 (0.95)	6 (2.86)
Prostate	4 (1.90)	0	4 (1.90)
Thyroid	1 (0.48)	2 (0.95)	3 (1.43)
Other cancer types	2 (0.95)	9 (4.29)	11 (5.24)
Time of diagnosis			
<12 months	54 (25.71)	127 (60.48)	181 (86.19)
13–24 months	5 (2.38)	1 (0.48)	6 (2.86)
25–36 months	2 (0.95)	7 (3.33)	9 (4.29)
37–48 months	4 (1.90)	3 (1.43)	7 (3.33)
49–60 months	0	2 (0.95)	2 (0.95)
>60 months	0	1 (0.48)	1 (0.48)
Unknown	3 (1.43)	1 (0.48)	4 (1.90)
Cancer stage			
Stage 1	24 (11.43)	27 (12.86)	51 (24.29)
Stage 2	14 (6.67)	65 (30.95)	79 (37.62)
Stage 3	10 (4.76)	22 (10.48)	32 (15.24)
Stage 4	20 (9.52)	28 (13.33)	48 (22.86)
Type of intervention during the past 6 months			
Surgical	1 (0.48)	8 (3.81)	9 (4.29)
Chemotherapy	22 (10.48)	41 (19.52)	63 (30.00)
Radiotherapy	24 (11.43)	21 (10.00)	45 (21.43)
Surgical + chemotherapy	8 (3.81)	57 (27.14)	65 (31.0)
Surgical + radiotherapy	7 (3.33)	7 (3.33)	14 (6.66)
No treatment	6 (2.86)	8 (3.81)	14 (6.66)

Most of the patients were taken care of by their first‐degree relatives in the past 30 days, while 10% of the patients did not have a caregiver at home and had to manage their own care (Table 3). None of the patients received professional home health care services.

**TABLE 3:** Home care uses during the previous 30 days.

Home care usage	Female	Male	Total
Home care provider			
First degree relative	118 (56.19)	64 (30.48)	182 (86.7)
Other relative/ friend	5 (2.38)	2 (0.95)	7 (3.3)
Other caregiver	0 (0)	0 (0)	0 (0)
None	19 (9.05)	2 (0.95)	21 (10.0)
Professional home health care			
Received	0 (0)	0 (0)	0 (0)
Not received	68 (32.4)	142 (67.6)	210 (100)

The overall mean score for functional scales was higher for physical functioning (54.92) and lower for social functioning (38.89) (Table 4). The highest score on the symptom scale was for financial problems (63.02), while the lowest was for diarrhea (33.01). Men and women aged 18–39 years were found to have the lowest functional scores (30.00 and 35.63) on the social functioning scale, although men in the same age range had the highest cognitive function scores (70.00). Women over 70 years of age had the highest overall scores on the cognitive function scale (66.67). The symptom scale revealed high scores for financial difficulty (63.02), appetite loss (57.46), and nausea and vomiting (52.70), indicating that these were the most prevalent symptoms reported by the patients. Men aged 40–49 years reported worst symptoms for nausea and vomiting (75.00), while women aged 50–59 years suffered from appetite loss (65.81). With a low mean score of 33.01 for diarrhea and 33.97 for constipation, respectively, these symptoms appeared to be the least prevalent for both sexes in the present study. Men aged 40–49 years scored the lowest on the symptoms scale for constipation (8.33) whereas women aged 60–69 years scored the lowest on the symptoms scale for diarrhea (13.89). The total score for global health status and QoL was 47.98, which was higher for women than for men (49.41 vs. 44.98).

**TABLE 4:** EORTC QLQ‐C30 reference values for the study population by age and sex.

Scale	Sex	Age range (in years)					Total	
		18–39	40–49	50–59	60–69	>70		
		Mean (SD)	Mean (SD)	Mean (SD)	Mean (SD)	Mean (SD)	Mean (SD)	
Functional scales								
Physical	Male	60.00 (20.12)	54.17 (12.57)	55.56 (16.90)	58.33 (22.15)	42.22 (14.14)	55.10 (18.67)	54.92 (18.37)
	Female	57.47 (19.95)	53.98 (17.11)	53.85 (18.31)	49.21 (15.84)	64.44 (20.37)	54.84 (18.30)	
Role	Male	46.67 (17.21)	54.17 (23.14)	50.79 (22.03)	55.83 (24.34)	42.59 (32.40)	50.98 (23.54)	54.52 (22.22)
	Female	54.60 (18.30)	57.32 (22.38)	56.84 (19.39)	54.76 (22.45)	56.94 (31.35)	56.22 (21.43)	
Emotional	Male	36.67 (24.28)	39.58 (18.77)	44.05 (17.90)	52.92 (25.11)	35.19 (21.15)	43.87 (22.10)	43.97 (20.74)
	Female	43.68 (19.75)	44.31 (20.36)	42.09 (20.23)	42.86 (15.65)	52.08 (27.32)	44.01 (20.13)	
Cognitive	Male	70.00 (21.94)	47.92 (24.30)	59.52 (28.66)	44.17 (22.47)	38.89 (20.41)	52.45 (25.96)	53.33 (24.17)
	Female	49.43 (18.62)	55.69 (24.90)	50.00 (25.93)	55.56 (22.57)	66.67 (17.41)	53.76 (23.35)	
Social	Male	30.00 (26.98)	39.58 (21.70)	32.54 (20.05)	41.67 (23.25)	31.48 (28.19)	35.53 (23.20)	38.89 (24.85)
	Female	35.63 (20.76)	41.06 (25.30)	39.74 (25.82)	46.83 (25.07)	41.67 (36.58)	40.49 25.53)	
Symptom scale								
Fatigue	Male	41.11 (19.63)	41.67 (25.02)	40.21 (14.26)	43.33 (23.05)	39.51 (15.82)	41.34 (18.99)	45.18 (19.09)
	Female	47.51 (19.22)	49.86 (17.76)	46.15 (18.12)	41.80 (17.88)	48.15 (26.52)	47.03 (18.93)	
Nausea and vomiting	Male	43.33 (29.61)	75.00 (34.50)	54.76 (29.88)	50.83 (27.29)	48.15 (32.75)	53.43 (30.41)	52.70 (29.09)
	Female	54.60 (28.83)	53.25 (28.68)	47.44 (28.23)	51.59 (26.82)	61.11 (32.82)	52.35 (28.53)	
Pain	Male	31.67 (19.95)	45.83 (17.25)	50.79 (24.42)	59.17 (21.27)	33.33 (16.67)	47.54 (23.08)	48.97 (23.06)
	Female	46.55 (21.54)	50.81 (26.60)	52.99 (20.90)	42.06 (20.83)	55.56 (23.92)	49.65 (23.10)	
Dyspnea	Male	50.00 (28.32)	50.00 (35.63)	49.21 (30.95)	46.67 (33.16)	62.96 (35.14)	50.49 (31.80)	44.60 (30.33)
	Female	42.53 (23.40)	34.96 (26.82)	43.59 (32.58)	52.38 30.86)	38.89 (34.33)	41.78 (29.29)	
Insomnia	Male	46.67 (23.30)	54.17 (17.25)	47.62 (22.54)	38.33 (24.84)	48.15 (17.57)	45.59 (22.23)	44.60 (25.98)
	Female	44.83 (28.56)	47.15 (26.85)	38.46 (25.98)	49.21 (30.95)	41.67 (28.87)	44.13 (27.66)	
Appetite loss	Male	70.00 (29.19)	54.17 (24.80)	61.90 (26.43)	51.67 (38.20)	51.85 (37.68)	57.84 (31.87)	57.46 (30.94)
	Female	52.87 (31.52)	56.10 (30.22)	65.81 (28.08)	60.32 (30.95)	38.89 (31.25)	57.28 (30.59)	
Constipation	Male	16.67 (28.33)	8.33 (23.57)	26.98 (30.95)	35.00 (36.63)	55.56 (33.33)	29.41 (33.84)	33.97 (33.88)
	Female	34.48 (35.05)	31.71 (33.29)	39.32 (34.94)	49.21 (32.69)	22.22 (25.95)	36.15 (33.80)	
Diarrhea	Male	13.33 (23.31)	25.00 (29.55)	26.98 (34.35)	30.00 (26.27)	25.93 (32.39)	25.49 (29.43)	33.01 (31.78)
	Female	36.78 (31.30)	39.84 (29.08)	35.90 (34.53)	44.44 (35.49)	13.89 (26.43)	36.62 (32.33)	
Financial difficulty	Male	70.00 (29.19)	66.67 (30.86)	58.73 (29.64)	56.67 (36.03)	51.85 (29.40)	59.80 (31.31)	63.02 (32.32)
	Female	65.52 (30.19)	68.29 (30.69)	65.81 36.26)	53.97 (32.45)	63.89 (36.12)	64.55 (32.79)	
Overall								
Global health status/QoL	Male	35.83 (25.17)	50.00 (16.67)	40.08 (18.93)	45.83 (21.54)	60.19 (14.30)	44.98 (20.77)	47.98 (20.38)
	Female	48.85 (19.38)	53.25 (18.25)	47.65 (19.59)	50.00 (19.72)	42.36 (29.40)	49.41 (20.11)	

When the QoL was evaluated with EORTC‐QLQ‐C30 score it was observed that scores for the functional scales decreased with advancing cancer stage. When symptoms were evaluated, fatigue, nausea and vomiting and pain worsened during the initial stage of the cancer but were less intense during later stages. Dyspnea and insomnia worsened with the advancing cancer stage (Table 5).

**TABLE 5:** EORTC‐QLQ‐C30 scores according to cancer stage.

Scale	Stage 1 (*n* = 51)	Stage 2 (*n* = 79)	Stage 3 (*n* = 32)	Stage 4 (*n* = 48)	*p*‐Value
	Mean (SD)	Mean (SD)	Mean (SD)	Mean (SD)	
Functional scales					
Physical	65.10 (18.46)	55.36 (17.52)	51.87 (17.33)	45.42 (14.85)	<.001
Role	62.09 (21.10)	54.64 (22.48)	54.17 (22.00)	46.53 (20.90)	.042
Emotional	50.82 (16.77)	45.68 (22.60)	41.93 (20.68)	35.24 (18.61)	.008
Cognitive	57.52 (25.45)	56.75 (24.54)	49.48 (19.16)	45.83 (23.70)	.020
Social	40.20 (26.49)	41.35 (25.30)	36.98 (27.67)	34.72 (20.00)	.483
GH/QoL	36.76 (24.62)	49.47 (20.43)	50.26 (16.19)	55.90 (11.40)	.001
Symptom scales					
Fatigue	49.46 (18.37)	47.96 (18.79)	46.18 (20.18)	35.42 (16.63)	.144
Nausea and vomiting	53.59 (28.35)	54.22 (30.37)	51.04 (27.41)	50.35 (29.47)	.367
Pain	59.80 (23.14)	48.10 (24.02)	46.35 (19.74)	40.63 (19.42)	.053
Dyspnea	37.25 (24.63)	35.44 (28.42)	48.96 (29.31)	64.58 (30.29)	<.001
Insomnia	40.52 (26.93)	46.41 (27.95)	40.62 (23.55)	48.61 (22.76)	.509
Appetite loss	50.33 (32.91)	55.27 (30.61)	58.33 (31.68)	68.06 (26.59)	.391
Constipation	24.84 (32.55)	34.18 (32.46)	45.83 (32.52)	35.42 (36.65)	.067
Diarrhea	25.49 (30.98)	38.82 (33.09)	34.37 (29.92)	30.56 (30.62)	.372
Financial difficulty	57.52 (33.39)	61.18 (35.18)	61.46 (29.46)	72.92 (26.32)	.239

## 4 DISCUSSION

The present study has reported a high number of women cancer patients in Bangladesh and revealed that the majority were married Muslim by religion, of non‐residential status in Dhaka. Breast cancer was the most prevalent cancer, followed by cancer of the uterus‐cervix‐vagina and gallbladder‐bile duct‐pancreas. Cancer is a noncommunicable disease that generally affects the middle and older age population, as seen in this study. However, the study reported 18.6% of cancer patients in the age group18–39 years. The Global Burden of Disease (GBD) Study in 2019 estimated that cancer was the fourth leading cause of death and the tenth leading cause of disability adjusted life years (DALY) in young adults and adolescents.^10^

The prevalence of cancer is different for men and women in Bangladesh. Lung and oral cancer are the most prevalent cancers in males, with 41% of men aged 15 and older being smokers compared to 1.8% of women.^5^ There are 20 million individuals who use tobacco in some way, among which 1 in every 5 tobacco user is a woman.^5^ Every year, diseases linked to tobacco use claim the lives of 57 000 people.^5^ In contrast breast and cervical cancer are more prevalent in women,^5^ and the majority of the patients in the present study had come to the hospital for cancer care. The incidence rate of breast cancer in Bangladesh in 2020 was 22.5 per 100 000 women, with a mean age of patients being 41.8 years, and 90% of the diagnoses were in the advanced stage of the disease.^11^

Some published literature discusses the reluctance and hesitancy of Bangladeshi women to seek medical care, especially for breast or cervical cancer, which are regarded as taboo, leading them to avoid medical care.^2^, ^11^ The lack of health screening programs, knowledge of self‐screening (breast self‐examination), poverty, illiteracy and inaccessibility to health services in rural areas were among the other contributing factors to worsen symptoms before diagnosis.^11^, ^12^ The government of Bangladesh made human papilloma virus (HPV) screening services available for free to all women aged 30–60 years in all districts.^13^ This initiative could have contributed to an increase in the number of female patients in the present study, likely due to improved case detection resulting from screening.

In the present study, all functional scores were lowest for the male cancer patients older than 70 years. However, in women, functional scores in the advancing age group improved compared to the 18–39‐year age group, indicating that women cope better than men as age advances. Younger women experience more psychological morbidity and lower QoL after breast cancer diagnosis than older women, as older patients are believed to have greater social and emotional functioning, which has been associated with better QoL, while younger patients have poor adjustment, intolerable treatment, less coping, and significant distress.^14^ In the present study, female patients had the lowest mean social function score among the five function scales, indicating poor social support for women in the family and society. Studies from Saudi Arabia and Malaysia revealed that social support from family and society improved the general QoL for female breast cancer patients.^15^, ^16^

Our findings indicate that Bangladeshi female cancer patients had higher QoL scores than male patients in all functional subscales, except physical function. This contrasts with QoL studies in European countries (Austria, Germany, Slovenia, Denmark, Sweden, France, Hungary, Italy, Poland, Netherlands, Turkey, Russia, United Kingdom), Canada, and the United States, where men had a higher functional subscale score and lower burden of symptoms than women.^17^, ^18^, ^19^, ^20^, ^21^, ^22^, ^23^, ^24^

The function scale scores decreased as the cancer stage advanced, except for social function, which increased from stage 1 to stage 2 and then decreased. General health improved as the cancer advanced. When we evaluated symptoms and single‐item scales, we found that financial difficulties were the main problems, followed by appetite loss, nausea and vomiting, and pain. This differs from the experience of Tanzanian cancer patients, who suffered more from pain and insomnia, and Iranian cancer patients, who presented with gastrointestinal symptom burden like nausea/vomiting and diarrhea.^25^, ^26^

The overall QoL of cancer patients in the present study was 47.98 which was 45.71 for males and 49.10 for females. According to the EORTC scoring guidelines, a high QoL is indicated by high scores on the functioning and global health status/QoL scales (0–100), while a high burden of symptoms is indicated by high scores on the symptom scales.^8^ However, it is not clear what the manual's cutoff score should be. A cutoff score of 75 was utilized in an Ethiopian study to determine whether the function and symptom scores indicated an affected or unaffected status.^27^ In contrast, an Indian study utilized a cutoff score of 50 to assess the QoL.^28^ Selecting a cutoff point by the researcher is debatable and not without bias.^29^ Based on the cutoff score of 75, the QoL in our population is severely affected. Cancer patients in Bangladesh report lower QoL, more symptoms, and a greater need for financial assistance and pain management like cancer patients in Ethiopia.^27^, ^30^ Global health and QoL were higher in developed European countries such as Austria (75.65), Germany (67.0) and Italy (64.87) when compared to our study (47.98).^17^, ^31^, ^32^

In our study, the vast majority of cancer patients were cared for by first‐degree relatives or close friends. A Japanese study found that family members and caregivers had a pessimistic view of QoL of their patients, reflecting their own emotional discomfort and the stress of providing care.^33^ To minimize this bias, we encouraged patients to fill out the questionnaire by themselves if they were able to read and write. For illiterate patients, we dictated the items in the questionnaire and recorded their responses ourselves. This was a time‐consuming process, but it allowed patients to express concerns about their QoL that they might not have otherwise acknowledged, and they could feel as though their concerns are legitimate and warrant further discussion.^34^

The QoL also depends on the stage of the cancer and the treatment modality. There were 14 cases in the present study who were on palliative care. The main palliative care in cancer patients is pain management. The financial burden of cancer, which includes both direct and indirect costs such as missed wages, income, and savings, is a priority concern.^35^ A crucial part of cancer care should involve discussing the expense and benefit of cancer therapy, as well as the accessibility and availability of resources, to determine the financial strain.^36^ In a developed country like the United States, where more anti‐cancer drugs are available to choose from, many patients report that cancer care providers are not adequately concerned about their financial burdens.^35^ In a low‐income country like Bangladesh, the economic hardship generated by cancer treatment leads to inadequate treatment or treatment withdrawal, ultimately decreasing QoL. In our study, more than two‐thirds of the non‐Dhaka residents who participated had come to the capital for treatment, indicating a lack of adequate cancer treatment facilities in other parts of the country.^2^

However, there is still a need for a viable solution to this issue. The health insurance scheme, or financial aid for cancer patients if provided by the government, such as in neighboring countries like Nepal and India, could benefit many patients. The government can introduce a health insurance policy for the whole family with yearly renewal charges. The government could provide financial aid or subsidy to cancer patients yearly and also to elderly citizens aged 70 years or older, so that many patients would benefit and there will be fewer cases of discontinuation of treatment. The government can purchase the cancer medications and provide them in subsidy for the needy patients. Decentralizing more cancer care centers throughout the country will decrease the need of the patients to travel to the capital city Dhaka for their diagnosis and treatment, which would provide a relief to their financial burden. The other challenge facing cancer patients is the long wait time for appointments. There are increasing numbers of cases every year; however, there are limited hospitals dedicated to cancer treatment and lack of adequate oncologists in the country. There are around 300 medical oncologists in the entire country, which is the most densely populated country in the world. The government can provide sponsorship (scholarship) to the doctors who wish to pursue their career in cancer treatment but could not do so due to financial constraints.

Although cancer‐specific QoL studies have been reported from Bangladesh, QoL study in cancer patients irrespective of cancer type is rare. Therefore, this study is a great representation of a sample of Bangladeshi population in terms of QoL. Limitations of the present study are due to its cross‐sectional design, small sample size, and single study site. Furthermore, the QoL if assessed during diagnosis, after diagnosis and pretreatment and post‐treatment in the same patient, would allow a comparison of QoL over time. The present study included only adults aged 18 years and above; however, the QoL in cancer patients less than 18 years of age was not explored. There are limitations noted in the questionnaire itself. EORTC QLQ‐C30 does not provide a cutoff score and a clear interpretation of scores. Personal hygiene, fear, social and cultural factors are believed to have an impact on QoL in cancer patients, which was not explored in the present study.

The current study highlights the declining QoL of cancer patients. More emphasis is placed on vigorous treatment and cures in therapeutic practice, but patient comfort and quality of life receive the least attention. Additional research on QoL of cancer patients in Bangladesh could shed light on the burden of symptoms that negatively impacts QoL in general. To better understand how quality of life has altered over time, future research could be initiated to examine patient QoL before, during, and after treatment.

## 5 CONCLUSION

The EORTC QLQ C‐30 questionnaire was found to be easy to administer and helped patients feel their symptoms were being attended to. In general, the QoL of the female cancer patients in the study was better than that of the males. The financial burden was identified as a major factor that deteriorated the QoL in Bangladeshi cancer patients. However, when compared to the European and American countries, the overall QoL was poor, although it was similar to that of African countries. The study highlights the multiple challenges faced by cancer patients, including physical and emotional symptoms, financial difficulties, and lack of adequate support. These findings provide useful insights for developing targeted interventions to improve the QoL of cancer patients.

## AUTHOR CONTRIBUTIONS

**Nazmul Islam**: Conceptualization, data collection, methodology, investigation, resources, writing—review & editing. **Alok Atreya**: Conceptualization, methodology, formal analysis, investigation, visualization, writing—original draft, writing—review & editing. **Kazi Jashim Uddin**: Data collection, writing—review & editing. **Md. Rashed Kaiser**: Data collection, writing—review & editing. **Samata Nepal**: Methodology, formal analysis, visualization, writing—original draft, writing—review & editing. **Ritesh G. Menezes**: Formal analysis, supervision, visualization, writing—review & editing. **Savita Lasrado**: Supervision, writing—original draft, writing—review & editing. **Muhammad Abdullah‐Al‐Noman**: Resources, writing—review & editing.

## CONFLICT OF INTEREST STATEMENT

The authors have stated explicitly that there are no conflicts of interest in connection with this article.

## ETHICS STATEMENT

The study proposal and consent form were approved by the Ethics Committee of the Delta Medical College Hospital, Dhaka (DLMCH/IEC/2022/1).

## Data Availability

The data that support the findings of this study are openly available in “Dryad” at doi: https://doi.org/10.5061/dryad.pnvx0k6s4.^9^
